# Nutrition and metabolism in the rehabilitative phase of recovery in burn children: a review of clinical and research findings in a speciality pediatric burn hospital

**DOI:** 10.1186/s41038-015-0004-x

**Published:** 2015-05-28

**Authors:** Kathy Prelack, Yong Ming Yu, Robert L Sheridan

**Affiliations:** Shriners Hospitals For Children, Boston, Massachusetts USA

**Keywords:** Pediatric burns, Nutrition, Burn rehabilitation

## Abstract

During the rehabilitation phase of burn injury, patient care transitions from critical care medicine to restorative treatment strategies that encompass physical and occupational therapies, nutrition repletion, and psychosocial support for community reintegration. As pediatric burn patients undergo rehabilitation, nutrition assessment remains ongoing to define nutritional status and any alterations in metabolism that may take place. For some, a persistent hypermetabolic state appears evident, and weight loss may continue. The severity and duration however varies among patients. Many patients enter their rehabilitative phase with visible lean body mass depletion, and the focus of nutritional therapy for them shifts to replenishing nutritional status, while supporting rehabilitative efforts. Over the past decade, we have conducted studies on energy and protein metabolism, body composition, including bone mineralization, and general wellness in over 130 patients to better understand changes in metabolism and nutritional status during the rehabilitative phase of recovery. This abstract summarizes our findings.

## Review

### Introduction

During the rehabilitation phase of burn injury, patient care transitions from critical care medicine to restorative treatment strategies that encompass physical and occupational therapies, nutrition repletion, and psychosocial support for community reintegration. Typically, this rehabilitative phase of care begins once the patient has achieved 95% definitive wound closure, is spontaneously breathing, necessitates less nursing care for dressing changes, and requires extensive physical therapy to promote function. These patients are also no longer fully reliant on nutrition support for nutritional requirements. However, many remain at nutritional risk. For some, a hypermetabolic state persists, and weight loss continues [1]. Such patients require some degree of nutritional supplementation. Other patients, while no longer hypermetabolic, enter their rehabilitative phase with remarkable lean body mass (LBM) depletion [2]. The focus of nutritional therapy for them shifts to replenishing nutritional status, while supporting rehabilitative efforts along with energy expenditure, alterations in protein metabolism and body composition which manifest in growth failure have been identified. Continuous monitoring and reassessment of nutritional status with modifications in nutritional therapy as indicated can accommodate the unique yet diverse needs of this population and support their therapeutic goals for recovery.

This paper identifies the major nutritional concerns of pediatric burn patients in as they transition into the rehabilitative phase of care. The findings in this paper are based on the 20 years of research and clinical experience in a specialty pediatric burn center and are corroborated by research in the burn community. We have identified five major nutritional risk factors that require nutrition intervention among patients progressing into this next phase of care. These risk factors include accelerated energy and protein turnover, bone demineralization, diminished body cell mass, and delayed growth. Suggestions for therapeutic interventions are provided.

### Alterations in total energy expenditure and its components: continuation of increased energy requirements

Severely burned children are at risk for both acute and chronic malnutrition. Acute undernutrition results from decreased intake and inflammation that accompanies severe burn injury [3-6]. While resting energy expenditure (REE) is expected to normalize as wound healing progresses, elevations of 130%–145% above normal persist until the time of discharge [7,8]. Studies have predominately focused on a persistent inflammatory state as the cause of sustained catabolism; however, multiple facets of care can affect energy balance [9,10]. Our own clinical experience in rehabilitating patients who fail to gain weight has been that REE is not significantly increased. This indicates that factors other than a persistent hypermetabolic state can influence energy balance in these patients. To investigate this further, we measured total energy expenditure (TEE) and its main components, REE, and physical activity level (PAL) in ten rehabilitating pediatric burn patients. REE was measured by indirect calorimetry, and PAL was assessed using a physical activity monitoring device worn on the wrist or ankle for a 24-h period. TEE and REE were compared to gender-specific, age-, and weight-matched norms using the Dietary Reference Intakes (DRI) standards. Energy intake was calculated to determine energy balance. Table 1 shows energy variables for this group, REE represented on average 56% of TEE, which is comparable to reference norms (DRI), although it was as high 76% in the most sedentary child. Of note, REE as a percent of predicted basal metabolic rate (BMR) was on average low, (92% ± 25%). However, when viewing REE relative to LBM, the group as a whole had an REE that is slightly higher than the reference norm. When stratifying patients according to who gained versus lost weight, patients who lost weight had a mean REE/LBM ratio which was greater than the norm (53.4 vs 39.1). This ratio was a significant predictor of weight loss in our population (*r* = 0.62; *p* < 0.05) [11].

**Table 1:** **Measures of energy variables in ten rehabilitative burned children**

**Energy variable**	**Mean (SD)**
Energy intake (kcal/kg)	54.7 ± 20 (32.7–97.4)
TEE (kcal/kg)	66.2 ± 16 (46.8–94.4)
REE (kcal/kg)	35.8 ± 7.4 (24.5–27.6)
AEE (kcal/kg)	20.3 ± 17.8 (2.2–51.2)
REE/TEE (%)	55.7 ± 14.5 (30.1–76)
Energy balance (kcal/kg/day)	−9.8 ± 16.9 (−31.9–+13.9)
REE/BMR (%)	92 ± 24.9 (41–120)
AEE/TEE (%)	36.2 ± 30.2 (2–81)

Patients were measured following wound closure. Data is presented as mean and standard deviation (SD).

*TEE* total energy expenditure, *REE* resting energy expenditure, *AEE* activity energy expenditure, *BMR* basal metabolic rate [11].

Energy expenditure associated with physical activity is the second main contributor to TEE in this group. Mean activity energy expenditure was 36% ± 30% of TEE (Table 1). Mean PAL, determined by TEE/BMR, for the group was 1.7 ± 0.28 (range: 1.2–2.0), classifying the group as active. In fact, 6 out of 10 subjects were classified as active or highly active with PAL scores greater than 1.6. Although not all of these lost weight, they did have negative energy balance during the 7-day measurement period. Weight loss therefore can occur both because of increased REE and increased physical activity [11]. Measurement of REE is recommended in this phase to determine if patients are still hypermetabolic or have increased needs due to physical therapy. Patients who are at least 120% of their REE are likely still in their catabolic and will lose weight if not provided with adequate nutrition. Conversely, maintaining a positive energy balance is necessary to minimize weight loss and support increased physical activity. We recommend the use of activity monitor to help calculate the appropriate physical activity factor to assure that their total requirements are met. In the absence of this, our studies indicate that TEE averaged 65 kcal/kg for our population. This can be used as a minimal calorie target level for most patients (greater than 3 years of age) who enter the rehabilitative phase of care.

### Alterations in protein kinetics during the rehabilitative phase of care: continuation of increased protein needs

Increased protein turnover following severe burn injury is well established. The extent and duration of protein breakdown including its effect on body composition will dictate nutrition therapy goals during rehabilitation. Multiple studies have indicated that protein breakdown persists following wound closure and well into the rehabilitative phase. This is at a time when a shift towards anabolism is greatly needed for continued recovery and meeting of physical therapy goals [12-14]. Diminished LBM and muscle strength is pronounced even 6 months after burn injury in children [1,2]. Whether this is due to continuation of a catabolic state versus a lack of proper therapeutic intervention to satisfy protein requirements is unclear and may vary among patients.

Studies of protein turnover in our hospital before and after wound closure, and after discharge have given us some insight as to what protein requirements are for rehabilitative patients. In our population measures of protein turnover using the N15 glycine method, with added measures of 3-methyl-histidine as indicators of skeletal muscle mass indicate that while protein turnover remains high during all stages of care, the kinetics vary among phases as does the contribution of skeletal muscle to protein breakdown. Table 2 shows that there was no significant difference between protein synthesis and protein breakdown with wound closure. However, while protein breakdown remained elevated, muscle breakdown dropped from 1.1 to 0.6 g/kg with wound closure (*p* < 0.0001), representing a decrease in the contribution of muscle protein to whole-body protein breakdown from 20% to 7%. Those patients who returned for a third measurement after discharge still demonstrated increased protein turnover. In fact, there was a positive trend towards increased turnover, synthesis, and balance, portraying an anabolic state. And although protein breakdown that was high, skeletal muscle protein breakdown returned to normal, and was consistent with 3MH values reported in healthy children. Analysis of dietary protein intake and its effect on protein kinetics indicates a difference in protein handling during the acute versus rehabilitative phase. During the acute phase, protein intakes of greater than 2.5 g/kg maintained positive protein balance via improved protein synthesis. During rehabilitation, a high-protein diet actually diminished protein breakdown, which normally cannot be achieved during a catabolic state. The potential for anabolism with a high-protein diet (similarly, 2.5 g/kg or more) exists [14].

**Table 2:** **Protein intake, protein turnover, and muscle protein breakdown**

**Phase**	**Protein intake g/kg/d**	**Protein turnover g/dk/d**	**Protein breakdown g/kg/d**	**Protein synthesis g/dk/d**	**Protein balance g/kg/d**	**Muscle breakdown g/kg/d**	**Muscle loss g/kg/d**
Acute phase (open wound)	3.0 (1.5)	369.4 (201.4)	9.5 (4.0)	12.2 (4.6)	2.8 (1.5)	20.2 (30.4)*	1.0 (0.6)
High protein	4.2 (1.2)	289.2 (79.8)	10.7 (4.6)	15.1 (5.9)**	4.4 (1.4)***	11.7 (8.4)	1.0 (0.5)
Low protein	1.6 (1.0)	460.5 (215.3)	13.3 (4.1)	14.4 (4.0)	1.2 (1.3)	5.6 (4.2)	0.7 (0.4)
Rehabilitative phase (wound closure)	2.5 (1.1)	404.3 (207.5)	10.6 (3.9)	12.9 (3.9)	2.3 (1.3)	7.0 (5.7)	0.6 (0.4)
High protein	3.2 (0.4)	324.3 (129.0)	8.7 (2.6)**	11.7 (2.5)	3.0 (0.4)***	8.0 (6.7)	0.6 (0.5)
Low protein	1.6 (1.0)	460.5 (215.3)	13.3 (4.1)	14.4 (4.0)	1.2 (1.3)	5.6 (4.2)	0.7 (0.4)

Data are expressed as means (SD).

High protein: >2.75 g/kg. Low protein: <2.75 g/kg.

*Significantly different from B, *p* < 0.05; **significantly different from LP, *p* < 0.05; ***significantly different from LP, *p* < 0.000.

### Changes in body composition following burn injury: decreased body cell mass and bone mineral density

The inflammatory response, if long continued, creates substantial changes in many aspects of body composition including bone density, LBM, body cell mass, and hydration. Sustained losses in LBM and bone mineral density (BMD) following burn injury and the risk of fracture have been identified [15]. Early findings of growth impairment and increased incidence of fracture prompted most investigative efforts to target BMD and vitamin D metabolism in burned children [15-18]. BMD is reduced in children with burn injuries and can be seen within 8 weeks of injury and last for up to 5 years. Etiologic factors that may alter bone kinetics in burn injury include cytokine production, increased endogenous glucocorticoid synthesis, and lack of mobilization [19-22]. During the acute phase in which injury levels of 25- and 1,25-hydroxyvitamin D are low, with a slow migration towards normal over time that correlates with a decline in inflammatory markers and subsequent recovery [23], aberrations in vitamin D status are difficult to correct, and serum levels are only marginally responsive to vitamin D supplementation thereby significant osteopenia can take place. More research is needed for appropriate dose and timing of vitamin D therapy [17,24]. However, in the interim, identifying patients who might be at higher risk can prompt interventions during rehabilitative care. We recently reviewed our BMD in our patients. Of 65 patients who had their bone mineral density assessed during rehabilitation (mean age and burn size was 10.2 ± 4.7 years and 46% ± 21.2% total body surface area (TBSA) respectively), *z*-scores averaged −0.93 ± 1.14, with a range of 1.6 to (−)3.3. Fifty-two percent of the patients had *z*-scores less than −1.0, requiring additional follow-up or intervention. Only ten patients (15.4%) had *z*-scores below (−)2.0. While their mean burn size was not significantly different from the rest of the group, seven of these were classified as otherwise malnourished. Diagnosis of malnutrition increased odds of having a bone mineral density *z*-score <−1.0 by 3.5 times (1.128, 10.916). TBSA, time of admission, and length of stay did not predict a low *z*-score. The findings emphasize the importance of reassessing nutritional status with transition to the rehabilitative phase of care [25].

**Figure 1:**
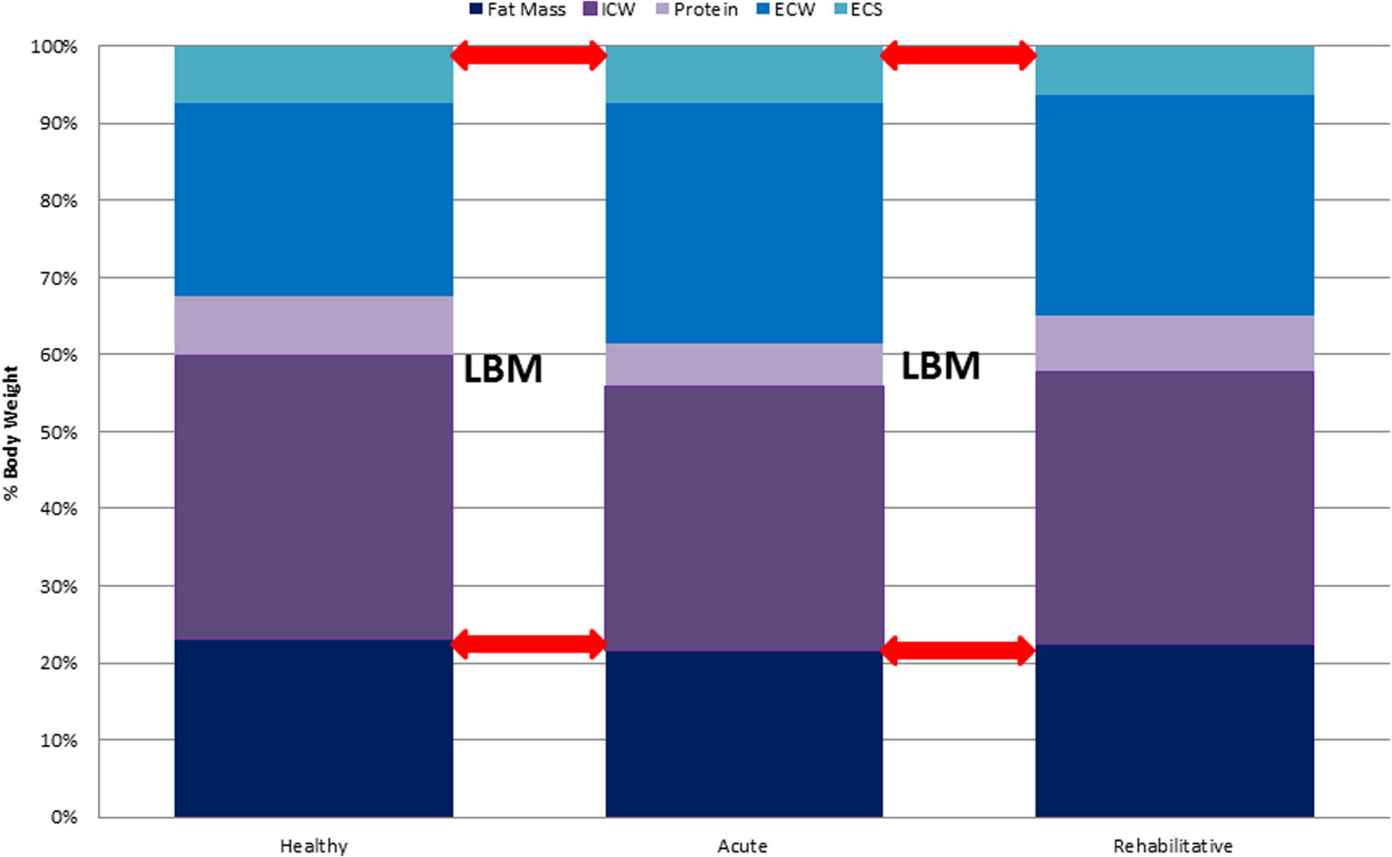
Changes of body composition during health and the acute and rehabilitative phases of burn injury. Body composition and its components are shown during health and the acute and rehabilitative phases of care. LBM = lean body mass; ICW = intracellular water; ECW = extracellular water; ECS = extracellular solid. Throughout these phases, overall lean body mass can go unchanged; alterations in water and protein components however take place.

With the exception of bone mineral density, little work has been done looking at body composition and its change following burn injury. Because significant depletion of the LBM and its components has a direct, negative impact on functional capacity and growth, losses of LBM are concerning. During metabolic stress, adipose tissue losses account for approximately one half of the weight loss experienced (Figure 1). The other half occurs within the body cell mass (BCM) component of the LBM, where net protein breakdown results in erosion of muscle. Hydrational changes also occur with an increase in extracellular water (ECW) and decrease in intracellular water (ICW), a proxy for BCM. This gain in ECW often masks the losses in ICW that occur as the BCM deteriorates. Because the expansion in ECW accompanies the diminution of the BCM, changes in LBM are often unremarkable. Body composition techniques such as dual-energy X-ray absorptiometry (DXA) that only measure LBM may not detect the severity of deterioration within the BCM. Alterations in hydration and their effects on the composition of LBM make it necessary to include measures of ECW and ICW when assessing change in body composition during acute recovery from severe burn injury. Measuring their changes in various tissues over the course of burn injury can identify those patients at extremely high risk for wasting and may serve as a better means of assessing response to nutritional therapy. This can help avoid significant wasting and malnutrition later on the rehabilitative phase of care. In our hospital, using stable isotope methodology, we are able to monitor changes in total body water (TBW) and its compartments throughout the different phases of care. During the acute phase of recovery, ICW losses were used to estimate body cell mass. ICW averaged (mean ± SD) 2.2 ± 2.0 L (*p* = 0.02) or 18.5% ± 0.4% of total body water. During the rehabilitative phase, mean ICW increased by 3.4 ± 3.7 L or 31.9% ± 14% of total body water, indicating an anabolic state. Furthermore, with rehabilitation, the ECW/ICW ratio decreased from 1.20 ± 0.14 L to 0.86 ± 0.20 L, due to a recoup of ICW and continued ECW losses. The importance of these is twofold: they indicate that fluid mobilization can extend into the rehabilitative phase of care; hence, ECW changes may confound LBM estimates by DXA and weight loss early on may be related to continuation of ECW. However, they also indicate that a recoup of ICW and presumably BCM mass can take place during the rehabilitative phase of care. Hence, opportunity exists to try to utilize this time for optimal nutritional rehabilitation [26].

### Impaired restoration of growth following severe burn injury and its implications

The above data indicates that despite wound closure, rates of protein breakdown and rates of skeletal muscle protein breakdown remain higher than rates among healthy children without burns for some time during their initial hospital stay. Subsequent losses in body cell mass may not be recovered for some time following the initial injury [26]. These metabolic aberrations can delay recovery and growth during convalescence. Furthermore, the physiological stress of frequent hospitalizations, repeated surgical procedures, and lingering physical impairments that accompany severe burn injury places burned children at continued nutritional risk. Chronic malnutrition, with growth faltering, is a consequence of these unrelenting environmental stresses.

Several studies have reported growth impairment in burned children, where there is a significant lapse particularly in height during the first year post-burn, which can resolved slowly over anywhere between 3 and 5 years post-burn [27-29]. It is clear that there is a delay in the onset of catch-up growth in burned children, a possibility that catch-up growth does not occur. In our series of 159 burned children, we monitored growth in height and weight using standardized units or *z*-scores. The use of *z*-scores, which enable comparisons of each child’s individual measure against the mean in a normally distributed population, enabled us to observe patterns of growth among burn children with a large age range over time. A positive *z*-score suggests that the individual has a measure that is greater than the mean, whereas a negative *z*-score indicates that the measure falls below the mean. *z*-scores are also useful for identifying degree of risk. Typically, a *z*-score between 0 and −1.0 is low risk, −1 and −2 moderate risk, and −2 or greater as high risk. In our population, mean height for age *z*-scores declined at 6 months and remained below their expected baseline by 0.5 to 0.76 SD (or *z*-score) units for up to 5 years. The prevalence, duration, and degree of this compromised growth reflect the severity of burn injury. Children with massive burn injury (>50% TBSA) can have moderate growth impediment with both stunting (−1.0 *z*-score height/age) and mild underweight status (−0.5 *z*-score weight/age). The magnitude of this impediment represents a deficit in height ranging from 1.6 to 5.8 cm, which was never fully restored in this study population over a 10-year period (Table 3). Stunted growth as defined by less than 2 SD from the norm was seen in 11% of patients, a prevalence that is higher than the expected norm of 2% [29].

**Table 3:** **Growth parameters for a semi-Longitudinal series of 159 children over a 10 year period**

**Post-injury time interval (** ***y*** **)**	**Height for age** ***z*** **-score**	**Weight for age** ***z*** **-score**
Admission (*n* = 159)	−0.23* (*p* = 0.02 )	+0.49
0.5 (*n* = 75)	−0.71**	−0.12**
1 (*n* = 110)	−0.58**	−0.14**
3 (*n* = 81)	−0.47** (*p* = 0.02)	−0.006**
5 (*n* = 65)	−0.51** (*p* = 0.006)	−0.03**
8 (*n* = 48)	−0.38*	+0.19**
10 (*n* = 21)	−0.13	+0.23

*Significantly lower than the reference standard; *p* < 0.0001, for all subsequent intervals unless otherwise shown; **significantly lower than the initial admission value; *p* < 0.0001, unless otherwise shown.

While a lapse in height is revered as a more significant chronic effect of poor nutrition, weight loss or lack of weight gain is compelling in burned children as well. Mittendorfer et al. in children under 8 years of age demonstrated continued weight loss for up to 4 years after discharge [30]. In extreme cases, where environmental conditions add further insult, stimulating growth can be extremely challenging despite aggressive provision of calories [31]. Paradoxically, once weight gain does resume, a pattern of catch-up growth where a disproportionate recovery of weight over height is concerning [29]. Typically, catch-up of linear growth resumes once malnutrition is corrected and a patient achieves positive energy balance. However, hormonal signaling to the epiphyseal growth plate by persistently increased glucocorticoids may attenuate linear growth. Other hormonal aberrations such as diminished vitamin D levels, growth hormone insufficiency, and insulin resistance can be contributory towards insufficient linear growth response as well, despite energy intake sufficient for promoting weight gain. This can lead to a disproportionate deposition of fat mass [21]. In our own population, weight is generally restored during the first 18 months after discharge, while height is not [29]. Over the long term, an incongruent recovery of weight over height runs the risk of overweight status in burned children. This phenomenon is now being recognized and reported in the literature. In a review by Mayes et al., 16% of rehabilitative burned children admitted for elective surgery were overweight, and 24% were classified as obese, a prevalence that is significantly greater than the national standard for US children [32].

### Recommendations for nutritional care during the rehabilitative phase

#### Assessment of nutritional status

Both clinical experience and evidence show that nutritional status and requirements during burn rehabilitation are distinct from the acute phase of care. Therefore, it is recommended that all patients have a full nutritional reassessment completed when they enter their rehabilitative phase of care. This should include new measures of weight and standing height. Measures of height and weight should be plotted on standardized growth charts so that growth can be monitored over time upon return visits. Although recommended intakes for certain nutrients in absolute terms maybe similar between the two phases, the metabolism of these nutrients is changed. For example, it is well accepted that energy and protein requirements are increased in patients with large open wounds areas to compensate for heat and energy losses as well as to promote wound healing. Intuitively, therefore, once healing has occurred, the metabolic burden is decreased and goals for nutrient intake can be adjusted downward. This is somewhat indicated given the down-regulation of acute phase reactants, and a gradual return of metabolic rate, body core temperature, and heart rate towards normal, which is so often observed in most burn patients. However, not all patients accomplish a normal metabolic state early on in the rehabilitative phase. And for many other patients, energy needs due to increased physical activity are increased. Similarly, protein is needed to support the high protein turnover that continues on to this later stage of recovery and to minimize obligatory protein losses. When possible indirect calorimetry to determine REE and a 24-h urine for nitrogen balance is suggested, this will help distinguish which patients remain hypermetabolic (as evidenced by increased REE and high urinary urea nitrogen losses). Biochemical indices of C-reactive protein and pre-albumin are also useful for determining inflammation and nutritional adequacy. Because repletion of bone mineral density is such an important aspect of the rehabilitative phase of care, measure of bone mineral density is recommended at routine interval. Many patients are too unstable to have a measure of bone mineral density during the acute phase. Therefore, a baseline measurement should be obtained to determine a patient’s risk for osteopenia once they enter the rehabilitation phase.

#### Guidelines for nutritional management of the rehabilitating pediatric burn patient

Promoting a patient’s transition to oral intake is a reasonable objective during the rehabilitative phase; however, haste to do this further compounds the risk of inadequate intake. For patients with evidence of a continued hypermetabolic state (based on indirect calorimetry, protein breakdown, and biochemical parameters), intermittent nutritional support is suggested. These patients remain at high risk of continued weight loss and protein catabolism and should have their energy protein intake monitored to maintain positive balance. For those who are no longer hypermetabilic but highly active in physical therapy (2 or more hours/day), signs of negative energy and protein balance are first detected by weight loss or failure to gain weight. Other indications in this patient category are diminished muscle strength or physical capacity. Prolonged suboptimal nutrition during the rehabilitative phase may eventually delay discharge home or compromise long-term growth and recovery. To prevent this, the goals or expected outcome for nutrition care must change from one of preservation of body cell mass to one of repletion and restoration. Table 4 summarizes the metabolic features of the rehabilitative phase and corresponding nutrition intervention strategies.

**Table 4:** **Highlight of key metabolic processes requiring nutritional assessment and intervention during the rehabilitative phase of care**

**Nutrition state or metabolic condition**	**Characteristic during rehabilitation**	**Nutritional assessment**	**Nutritional intervention**
Protein synthesis and breakdown	Increased synthesis and breakdown. Skeletal muscle breakdown is normal. Exogenous protein can diminish protein breakdown rate.	Nitrogen balance	Provide 2.5 g/kg of protein to cover obligatory losses. Maintain nitrogen balance in the positive by 2 g protein/kg day.
		Pre-albumin, CRP	
		Weekly	
Energy expenditure	Resting energy expenditure declines for most patients. Increased total energy expenditure due to increased physical activity.	Indirect calorimetry	Hypermetabolic: REE × 1.2
			Normal REE/intensive physical therapy: REE × 1.5 or 65 kcal/kg (to meet increased needs with physical activity)
		Weekly	
Bone mineral density	Altered vitamin D metabolism and bed rest results in large majority of burn patients to have mild to moderate bone loss following severe burn injury. Malnutrition increases the odds of having severe bone depletion.	DXA every 6 weeks	For bone mineral density *z*-scores
			>−1.0: no intervention
		Weekly	<−1.0: vitamin D3/calcium supplementation
			4–8 years: 1,000 mg/600+ IU
			9–18 years: 1,500 mg/600+ IU
			<−2.0 as above with 0.1–0.2 mg/kg oxandrolone
Growth	Growth delay is apparent in children with massive burn injury, effecting height more than weight.	Height and weights	Energy and protein as above to promote age appropriate rate of weight gain.
		Weekly	

*REE* resting energy expenditure, *CRP* C-reactive protein, *DXA* dual-energy X-ray absorptiometry, *IU* international units.

Energy and protein targets should be based on these measures with adjustment to accommodate for increased physical activity and to promote anabolism. As suggested, 65 kcal/kg or 150% times the REE should accomplish this, as well as 2.5 g/kg protein. From there, close monitoring of weight and weekly nitrogen balance can be useful to assure that the patients are in positive protein balance. For patients with bone mineral density losses, calcium and vitamin D supplementation is implemented. In severe cases (*z*-score less than −2.0), oxandrolone therapy can be instituted or extended throughout their rehabilitative care.

While the importance of maintaining nutritional status cannot be understated, established goals can be accomplished with a different therapeutic approach—one that is focused on wellness and long-term outcome. Emphasis on promoting oral intake and reliance on nutrient dense foods with supplemental enteral support if necessary is recommended. Consistently achieving caloric requirement and appropriate weight status is important; however, strategies are developed to promote patient self-efficacy. Concomitantly, muscle strengthening and aerobic training in addition to functional physical therapy are emphasized. Patient education and consideration of an environment conducive to attainment of goals after discharge is another important aspect. A comprehensive multidisciplinary approach to wellness has been a successful approach in our hospital during the later stages of rehabilitation for our patients. This program encourages continuity of care into convalescence by focusing on nutritional and physical status as well as emotional and social health. Table 5 shows key aspects of our wellness approach to patient care in the rehabilitation phase. Each week a schedule is created with planned interdisciplinary interventions based on established goals for the patient. A team meeting for the patient resides at the end of the week to discuss progress and future goals or needs for the patients. The success of this program is monitored by measurable outcome parameters such as change in bone and lean body mass, aerobic capacity, patient knowledge, and outcome surveys.

**Table 5:** **Multidisciplinary wellness program used to achieve comprehensive rehabilitative outcomes in burned children**

**Wellness discipline**	**Desired outcome**	**Specific intervention**	**Frequency and duration**	**Measurements/measurement outcome**
Physical therapy	To increase lean body mass and muscle strength	Exercise and strength training	2–3 times weekly for 6 weeks	Comparisons to standardized scores
		• Biceps curl		
		• Aerobic/endurance test (6-min walk)		
	To improve endurance to aerobic exercise			
		• Fine motor skill (manual dexterity)		
Nutrition therapy	To improve nutritional status and reverse malnutrition	Nutritional education and supplementation	Weekly for 6 weeks	DXA
		• High protein		Weekly weights
		• High calcium		
	To provide education in nutrition and general health	• General healthy diet		Patient knowledge of goals
		• Nutrition wellness activities (games, cooking, snacks)		
		• Wellness garden		
Recreational and music therapy	To provide relaxation and stress management techniques	Music therapy	1–2 times weekly for 6 weeks	Patient outcome surveys
		Therapeutic dance		
		Wellness garden		
		Parent education		
Psychological/social support	To promote self-esteem and social competence	Therapeutic outings	Bimonthly or as able	Patient outcome surveys
		Community reintegration		
	To identify and establish community resources for continued wellness			

The wellness program is provided to patients as an outpatient services. Each discipline is responsible for intervention strategies defined and the required frequency and duration.

## Conclusions

The rehabilitative phase of burn care is unique both therapeutically and metabolically, and nutritional support of the burn patient in this phase requires a full reassessment of nutritional status and goals. This phase offers an opportunity to provide nutritional therapy in a way that not only promotes anabolism but also augments efforts made at rehabilitation from a physical therapy and emotional standpoint. In our hospital, a wellness approach to care that incorporates patient education and lifelong strategies to health has been successful.

## Abbreviations

BCM: Body cell mass
BMD: Bone mineral density
BMR: Basal metabolic rate
DRI: Dietary Reference Intakes
DXA: Dual-energy X-ray absorptiometry
ECW: Extracellular water
ICW: Intracellular water
LBM: Lean body mass
PAL: Physical activity level
REE: Resting energy expenditure
TBSA: Total body surface area
TBW: Total body water
TEE: Total energy expenditure
